# Thermal Stability of Octadecyltrichlorosilane and Perfluorooctyltriethoxysilane Monolayers on SiO_2_

**DOI:** 10.3390/nano10020210

**Published:** 2020-01-26

**Authors:** Xiangdong Yang, Haitao Wang, Peng Wang, Xuxin Yang, Hongying Mao

**Affiliations:** 1 Department of Physics, Hangzhou Normal University, Hangzhou 310036, Chinawanghtphy@163.com (H.W.); 2 Department of Applied Physics, College of Electronic and Information Engineering, Shandong University of Science and Technology, Qingdao 266590, China; phywangp@sdust.edu.cn

**Keywords:** thermal stability, self-assembled monolayers, desorption and decomposition, electronic structures

## Abstract

Using in situ ultraviolet photoelectron spectroscopy (UPS) and X-ray photoelectron spectroscopy (XPS) measurements, the thermal behavior of octadecyltrichlorosilane (OTS) and 1H, 1H, 2H, and 2H-perfluorooctyltriethoxysilane (PTES) monolayers on SiO_2_ substrates has been investigated. OTS is thermally stable up to 573 K with vacuum annealing, whereas PTES starts decomposing at a moderate temperature between 373 K and 423 K. Vacuum annealing results in the decomposition of CF_3_ and CF_2_ species rather than desorption of the entire PTES molecule. In addition, our UPS results reveal that the work function (WF)of OTS remains the same after annealing; however WF of PTES decreases from ~5.62 eV to ~5.16 eV after annealing at 573 K.

## 1. Introduction

Self-assembled monolayers (SAMs) have been the subject of intense studies due to their promising applications in microelectromechanicalsystems (MEMS), molecular electronics, wetting, lubrication, and corrosion inhibition [1,2,3,4,5]. The formation of SAMs on various kinds of substrates has been investigated intensively [6,7]. Generally speaking, the precursor molecules reacted with water and became hydrolyzed into an analogous silanol form which interacted with the hydrophilic surface layer to complete the formation of SAMs. Because of the strong interaction between the head group of precursor molecules and the substrates, a close-packed monolayer was fabricated. In addition, the physical and chemical properties of SAMs can be manipulated by simply choosing the terminal group of precursor molecules with different functionalities. For example, the methyl group of octadecyltrichlorosilane (OTS) reduced the susceptibility to stiction [8], and the trifluoromethyl group of perfluorodecyltrichlorosilane (FDTS) resulted in highly hydrophobic and oleophobic monolayers, which prevented the device from adsorption of ambient moisture and other materials [2,9].

An important consideration in the practical application of SAMs in MEMS devices was their thermal stability. It was necessary for the SAMs to withstand the high temperature that was commonly used during fabrication and packaging steps [10,11,12]. In this regard, extensive efforts have been devoted to study the thermal behavior of SAMs on different substrates. Thermal behavior of alkyl monolayers on silicon surfaces has been studied using high-resolution electron energy loss spectroscopy (HREELS) [13]. The monolayers were found to be stable up to ~615 K, and the decomposition of the monolayers took place when the annealing temperature increased to 785 K. In situ X-ray photoelectron spectroscopy (XPS) measurements have also been employed to investigate the thermal stability of FDTS on oxidized Si(100) surface. Evidenced by the constant CF_3_/CF_2_ peak area ratios during the annealing process, the desorption of FDTS occurred by the loss of the entire molecular chain [14]. On the other hand, HREELS results confirmed that the desorption of OTS was through C–C bond cleavage upon heating. Above 740 K, the alkyl chains began to decompose, resulting the desorption of hydrocarbon fragments [15,16]. These results indicated that the thermal behavior of SAMs was essentially determined by the terminal group of precursor molecules. Although investigations of the thermal stabilities of SAMs, including desorption, decomposition, odd-even effects, and alkyl chain length effects, have been performed [17,18,19], to date there isno study about the impact of thermal annealing on the work function (WF) of SAMs, which is of great importance in determining device performance [20]. 

In the present study, the thermal behavior of OTS and PTES on SiO_2_ substrates has been investigated by in situ UPS and XPS measurements. It is found that OTS is thermally more stable than PTES upon vacuum annealing. As evidenced by the slowly decreased normalized atomic ratio of C/Si and constant WF of OTS, no decomposition but desorption of physisorbed OTS precursor molecules occurs upon annealing. On the other hand, PTES decomposes at a lower temperature and the loss of fluorinated species occurs by decomposition of CF_3_ and CF_2_ species. A WF decrease of ~0.46 eV is also observed for PTES on SiO_2_ substrate after annealing.

## 2. Materials and Methods 

### 2.1. Preparation of Octadecyltrichlorosilane(OTS) and PTES on SiO_2_

The SiO_2_ substrate (Si(110) with native oxide layer)was firstly treated with O_2_ plasma for 15 min for a hydrophilic surface during the formation of OTS and PTES. After O_2_ plasma treatments, SiO_2_ substrates were immersed in piranha solution (3:1 sulfuric acid to hydrogen peroxide, HPLC grade, Fisher Scientific) for 20 min, and then thoroughly rinsing using DI water. Finally, they were dried by nitrogen flow. In the case of OTS (Sigma-Aldrich), the clean SiO_2_ substrate was placed in a sealed vial filled with 0.3 mL octadecyltrichlorosilane (OTS). The sealed vial with SiO_2_ substrate was heated to 120 °C and kept for 30 min under an argon atmosphere, followed by thoroughly rinsed with toluene, methanol, DI water, and dried by nitrogen flow. For the fabrication of PTES, the clean SiO_2_ substrate were placed in a sealed vial filled with 0.5 mL 1H, 1H, 2H, and 2H-perfluorooctyltriethoxysilane (PTES) (Sigma-Aldrich), and then the sealed vial with SiO_2_ substratesheated in an oven at 120 °C for 60 min. After functionalization, the SiO_2_ wafer was rinsed by toluene, methanol, DI water, and dried by nitrogen flow. Water contact angle measurements have been used to confirm the successfully prepared OTS and PTES on SiO_2_. The water contact angle is less than 10° for pristine SiO_2_ after O_2_ plasma treatments. The water contact angle is 103° ± 2° and 108° ± 3° for OTS and PTES, respectively. This is in good agreement with previous reported value for the water contact angle of OTS and PTES monolayer on SiO_2_ [8,21].

### 2.2. Characterizations

In situ UPS and XPS measurements were carried out in a multifunctional ultrahigh vacuum (UHV) VT-SPM system (Omicron Instruments for Surface Science). The base pressure in the analysis chamber was ~3 × 10^−10^ mbar. In the case of UPS measurements, He I (21.2 eV) was the excitation source. A −5 V sample bias was applied to measure the sample WF which is determined by the linear extrapolation of the low kinetic energy part of UPS spectra. In our experimental set-up, WF = E_k_ − 0.7 eV. Using an Al Kα source (1486.6 eV), XPS measurements were performed. Moreover, the binding energy of all UPS and XPS spectra was calibrated using a sputtered clean gold sample. Considering the damage induced by photoelectrons and secondary electrons as well as the degradation of organic monolayer at high X-ray fluence [22], the duration of each scan was limited to less than 3 min to minimize the damage. Sample was mounted with Ta strips to a sample stub containing a built-in resistive heater in the preparing chamber. Each annealing step consisted of raising the temperature to a target value with the ramp time ~15 min, and then they were kept for 30 min before cooled down to room temperature (RT) for UPS and XPS analysis. After annealing, it was transferred to analysis chamber without exposing to air. The annealing temperature was calibrated using a thermocouple attached near to the sample. The morphology changes of OTS and PTES before and after vacuum annealing ware investigated by AFM (Veeco D3000, tapping mode).

## 3. Results and Discussion

**Figure 1:**
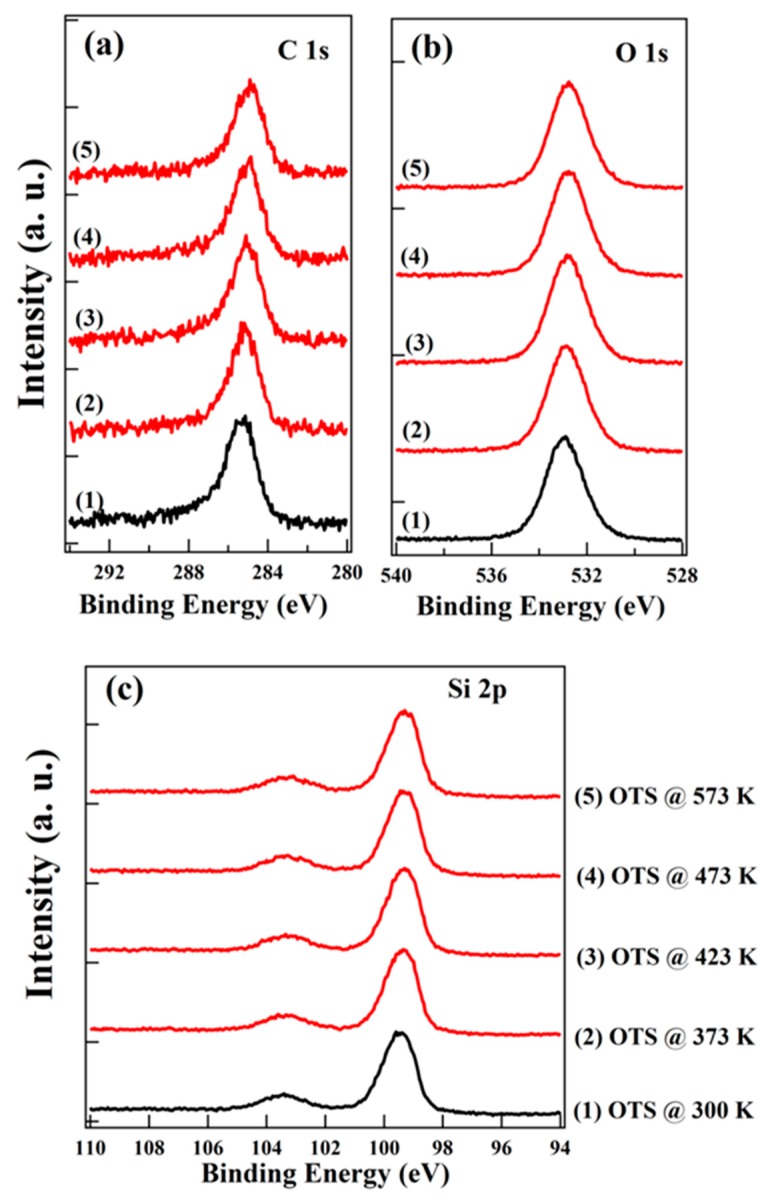
X-ray photoelectron spectroscopy (XPS) spectra of (**a**) C 1s, (**b**) O 1s, and (**c**) Si 2p for OTS as a function of vacuum annealing temperature.

Figure 1a–c shows C 1s, O 1s, and Si 2p XPS spectra as a function of vacuum annealing temperature for OTS assembled on SiO_2_, respectively. A single C 1s peak locating at ~285.3 eV can be identified, corresponding to the carbon atoms in the alkyl chain. The binding energy of C 1s remains essentially constant upon annealing to 573 K. Moreover, there is no change of C 1s peak shape upon annealing. The full width at half maximum (FWHM) of C 1s peak for pristine OTS is ~1.60 eV, and it is ~1.65 eV after annealing at 573 K for 30 min. For the O 1s and Si 2p XPS spectra, little change is observed after vacuum annealing until 573 K, including binding energy shift and peak broadening. Our XPS results for OTS before and after annealing indicate that the thermal stability of OTS is quiet good upon vacuum annealing up to 573 K. Previous reports based on HREELS and contact angle measurements have also revealed that self-assembled monolayers of alkyltrichlorosilane on oxidized Si (100) substrate can be thermally stable up to about 740 K in vacuum, and the thermal stability was independent of alkyl chain length [13,15]. The good thermal stability of OTS on SiO_2_ is also corroborated by our UPS measurements.

**Figure 2:**
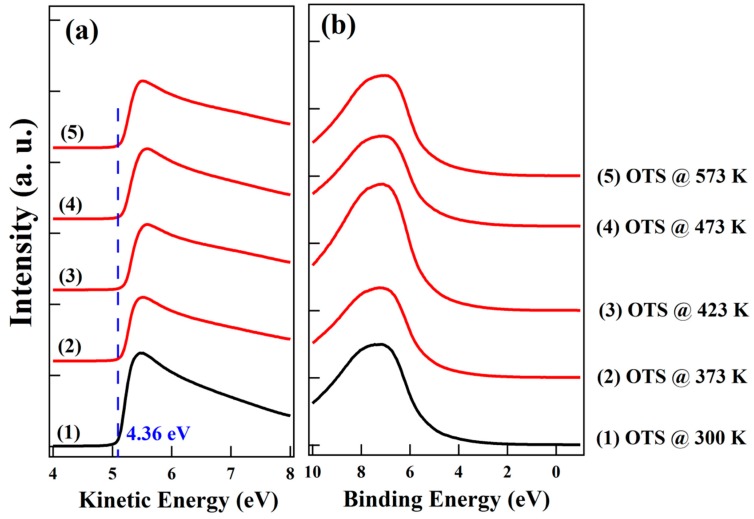
Spectra at the (**a**) secondary electron cut-off and (**b**) valence band region for OTS as a function of vacuum annealing temperature.

As shown in Figure 2a, the WF for pristine OTS is ~4.36 eV as measured from the linear extrapolation of secondary electron cut-off region. Upon vacuum annealing to 573 K, vacuum level shift is almost invisible, indicating that the WF of OTS remains the same after annealing. In addition, no unambiguous change of UPS spectra at valence band region for OTS upon annealing is observed as shown in Figure 2b.

**Figure 3:**
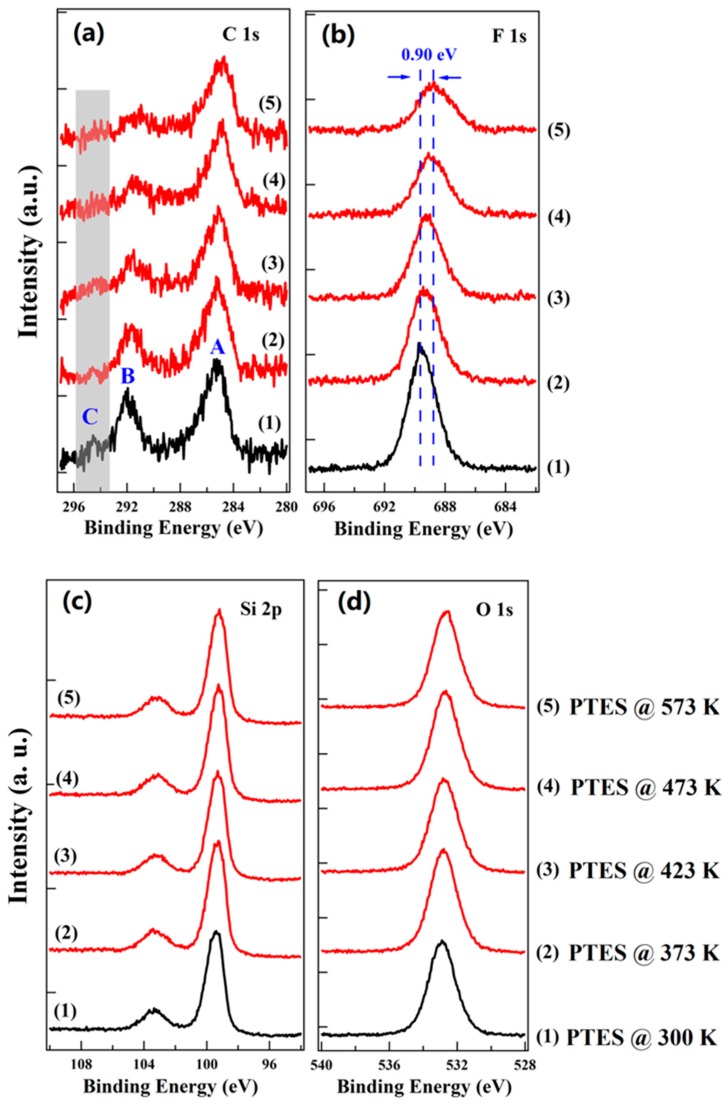
XPS spectra of (**a**) C 1s, (**b**) F 1s, (**c**) Si 2p, and (**d**) O 1s for PTES as a function of vacuum annealing temperature, respectively.

To reveal the effect of terminal groups on the thermal stability of SAMs, in situ UPS and XPS measurements are performed to investigate the thermal stability of PTES on SiO_2_ upon vacuum annealing. Figure 3 shows C 1s, F 1s, Si 2p, and O 1s XPS spectra as a function of annealing temperature for PTES assembled on SiO_2_. The C 1s region displays three main spectral features, marked by A, B, and C in Figure 3a. Peak A, located at ~285.3 eV, is rather broad with the FWHM of ~2.3 eV, deriving from carbon atoms of saturated hydrocarbon (CH_2_) and carbon atoms bonded to Si. Peak B, located at ~292.0 eV, is attributed to carbon atoms from CF_2_ species, and Peak C locating at ~294.3 eV derives from CF_3_ related carbon atoms [23,24]. The peak intensity of C 1s decreases with the increasing annealing temperature. Note that peak C can be still observed after annealing at 373 K, but it disappears when the annealing temperature is higher than 423 K which is highlighted in Figure 3a with grey color, suggesting the decomposition of CF_3_ species at relatively high temperature. 

**Figure 4:**
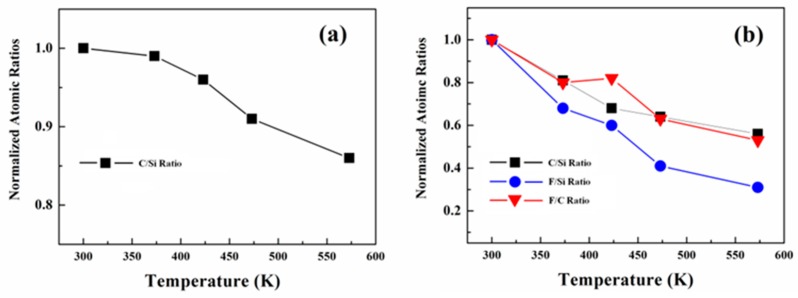
Atomic ratios determined from XPS of (**a**) OTS/SiO_2_ and (**b**) PTES/SiO_2_ samples as a function of vacuum annealing temperature.

By analyzing the normalized atomic ratio of C/Si, an in-depth understanding about the thermal behavior of OTS and PTES can be obtained. In the case of OTS, the normalized atomic ratio of C/Si for OTS decreases to ~86% after annealing at 573 K for 30 min, as shown in Figure 4a, indicating good thermal stability. On the other hand, the decomposition and desorption of PTES occurs even with moderate thermal annealing. As shown in Figure 4b, the normalized atomic ratio of C/Si for PTES decreases to ~81% and 68% after vacuum annealing at 373 K and 423 K, respectively. When the annealing temperature increases to 573 K, it further decreases to ~56%. Our findings demonstrate that the thermal stability of PTES is poorer than OTS. The F 1s region of pristine PTES on SiO_2_ is shown in Figure 3b, a single peak is observed locating at ~689.55 eV. Similar to C 1s peak, significant intensity attenuation of F 1s peak is observed after annealing to even moderate temperatures. The normalized atomic ratio of F/Si for PTES decreases to ~68% and 60% after vacuum annealing at 373 K and 423 K, respectively. As shown in Figure 4b, a further decrease to ~31% is found when the annealing temperature increases to 573 K. As the normalized atomic ratio of F/Si decreases faster than C/Si for PTES, the loss of fluorinated species are attributed to decomposition of CF_3_ and CF_2_ species rather than the desorption of the entire PTES molecule. This conclusion is further confirmed by the decreased normalized atomic ratio of F/C after annealing as shown in Figure 4b. Moreover, the F 1s peak shifts to lower binding energy part by ~0.90 eV after vacuum annealing at 573 K. This seems surprisingly, but it can be understood by considering the different molecular orientation of PTES after annealing [14]. Upon annealing and desorption of some of PTES molecules, the remaining PTES molecules tilt away from the nearly vertical arrangement of a well-packed monolayer [25], resulting in the observed lower binding energy shift upon annealing. More recently, orientation dependent electronic structures of organic molecules have also been demonstrated by some groups [26,27,28]. In addition, peak broadening of F 1s upon annealing has also been observed. The FWHM of F 1s peak for pristine PTES is ~2.05 eV, whereas it increases to ~2.70 eV after annealing at 573 K. Figure 3c,d shows the Si 2p and O 1s XPS spectra as a function of annealing temperature for PTES assembled on SiO_2_, there is no obvious change upon vacuum annealing until 573 K. 

**Figure 5:**
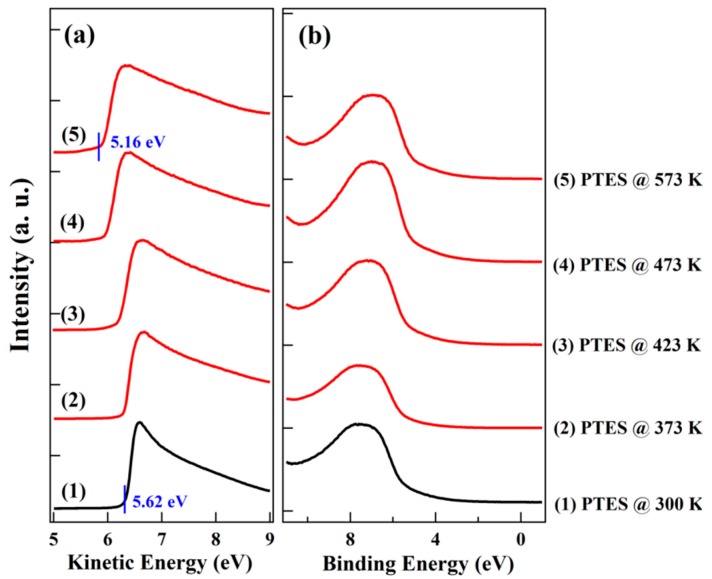
Spectra at the (**a**) secondary electron cut-off and (**b**) valence band region for PTES as a function of vacuum annealing temperature.

Figure 5 shows UPS spectra at the secondary electron cut-off and valence band region for PTES as a function of vacuum annealing temperature, respectively. The WF for pristine PTES modified SiO_2_ is ~5.62 eV, which is in good consistency with previous reports considering the high electron affinity of CF_3_ groups [24]. The high WF of ~5.62 eV for PTES modified SiO_2_ can be attributed to the dipole moment along the molecular axes of PTES. Due to the electron depleting characteristics of CF_3_ group, the dipole moment along the molecular axes is approximately −2.203 D for PTES [21]. The vacuum level (VL) shift after PTES modification can be estimated using the Helmholtz equation $\Delta V=\frac{\mu \cos\varphi }{\varepsilon \varepsilon_{0}}$ [29], where ΔV is the surface potential change and corresponds to a VL shift, μ is the dipole moment per unit area, and φ is the angle between the dipole and the surface normal. By assuming that the molecular density of monolayer PTES (N) is 1–2 × 10^14^ cm^−2^ and the effective dielectric constant (ε) inside the PTES monolayer is 2–3 [30], the VL shift is estimated to be 0.27–0.82 eV. As a result, the WF of PTES modified SiO_2_ is greatly increased. After vacuum annealing, a progressive vacuum level shift to lower kinetic energy part is observed, indicating the decrease of sample WF. When the annealing temperature increases to 573 K, the WF of PTES decreases to ~5.16 eV. On the basis of our XPS results, desorption and decomposition of PTES occur upon annealing. The decomposition of PTES molecule leads to lower dipole moment along the molecular axes, and the desorption of PTES molecule lead to lower molecular packing density, both of which decrease the VL shift according to Helmholtz equation. Therefore, a decreased WF of PTES modified SiO_2_ is observed upon vacuum annealing. By comparison with the negligible WF change for OTS after annealing, our UPS results support the conclusion that the thermal stability of PTES is poorer than OTS upon vacuum annealing until 573 K. Joëlle et al. has also observed the significant monolayer desorption of FDTS on oxidized Si(100) surface even when the temperature is lower than 300 °C, and the desorption mechanism followed first-order kinetics and was independent of the initial coverage [14].

**Figure 6:**
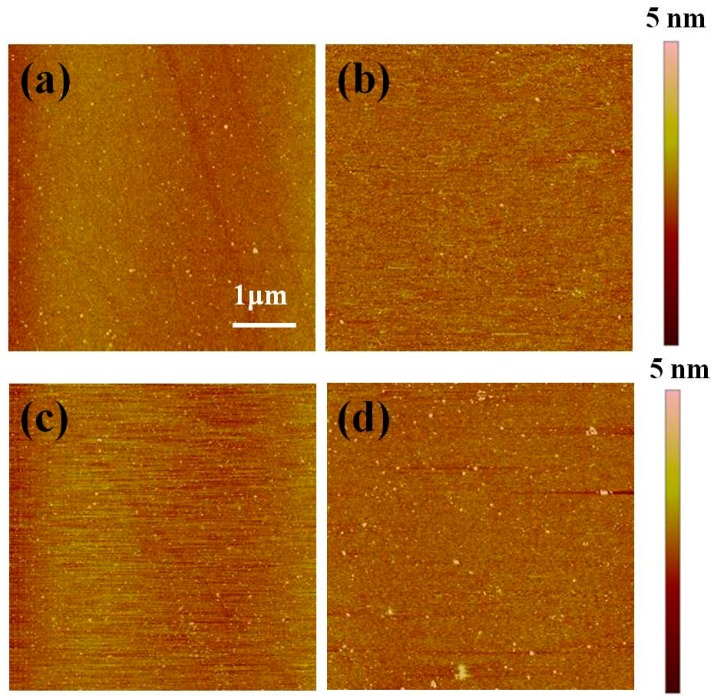
Images of OTS (**a**) before and (**c**) after vacuum annealing at 573 K; AFM images of PTES (**b**) before and (**d**) after vacuum annealing at 573 K.

Figure 6 shows AFM images of OTS and PTES before and after vacuum annealing at 573 K. All the AFM images are taken from the center of the samples. As shown in Figure 6a,b, there are no or only a few aggregations after the formation of OTS and PTES on SiO_2_ substrates. The root-mean-square (rms) surface roughness (R_q_) of pristine OTS and PTES is slightly lower than SiO_2_ substrates, and the R_q_ is ~0.36 nm, 0.19 nm, and 0.22 nm for SiO_2_ substrates, pristine OTS, and PTES, respectively. After vacuum annealing at 573 K, the R_q_ increases to ~0.25 nm for OTS, and only a few aggregations can be found as shown in Figure 6c. In the case of PTES, a larger increase of the R_q_ is observed which increases to ~0.43 nm after annealing at 573 K. Meanwhile, aggregations with larger size can be observed (Figure 6d), which could be the aggregation of molecule fragments followed by the decomposition of PTES upon annealing. Finally, we have also fabricated OTS and PTES on SiO_2_ with sub-monolayer coverage by controlling the formation time. Our XPS results (not shown here) confirm the conclusion that OTS is thermally more stable than PTES, which is independent of initial coverage.

## 4. Conclusions

We have investigated the thermal stability of OTS and PTES assembled on SiO_2_ substrates using in situ UPS and XPS measurements. Within different type of SAMs, the nature of the terminal group has a significant effect on its thermal stability. OTS is found to be stable up to 573 K with vacuum annealing, whereas PTES is less stable and starts decomposing at a moderate annealing temperature between 373 K and 423 K. XPS results of PTES before and after vacuum annealing show that the loss of fluorinated species occurs by decomposition of CF_3_ and CF_2_ species rather than desorption of the entire PTES molecule. In addition, our UPS results also indicate that the thermal stability of OTS is better than PTES upon annealing. The WF of OTS remains the same after annealing; however, the WF of PTES decreases from ~5.62 eV to ~5.16 eV after annealing at 573 K. Our results yield improved understanding of thermal behavior of OTS and PTES on SiO_2_ substrates and support the use of them for applications demanding high thermal stability.
